# Semi-Supervised Recurrent Neural Network for Adverse Drug Reaction mention extraction

**DOI:** 10.1186/s12859-018-2192-4

**Published:** 2018-06-13

**Authors:** Shashank Gupta, Sachin Pawar, Nitin Ramrakhiyani, Girish Keshav Palshikar, Vasudeva Varma

**Affiliations:** 1 Information Retrieval and Extraction Laboratory, Kohli Center for Intelligent Systems, International Institute of Information Technology, Hyderabad, India; 2 Tata Consultancy Services (TCS) Research, 54-B, Hadapsar Industrial Area, Pune, India

**Keywords:** Pharmacovigilance, Semi-supervised learning, Recurrent neural networks

## Abstract

**Background:**

Social media is a useful platform to share health-related information due to its vast reach. This makes it a good candidate for public-health monitoring tasks, specifically for pharmacovigilance. We study the problem of extraction of Adverse-Drug-Reaction (ADR) mentions from social media, particularly from Twitter. Medical information extraction from social media is challenging, mainly due to short and highly informal nature of text, as compared to more technical and formal medical reports.

**Methods:**

Current methods in ADR mention extraction rely on supervised learning methods, which suffer from labeled data scarcity problem. The state-of-the-art method uses deep neural networks, specifically a class of Recurrent Neural Network (RNN) which is Long-Short-Term-Memory network (LSTM). Deep neural networks, due to their large number of free parameters rely heavily on large annotated corpora for learning the end task. But in the real-world, it is hard to get large labeled data, mainly due to the heavy cost associated with the manual annotation.

**Results:**

To this end, we propose a novel semi-supervised learning based RNN model, which can leverage unlabeled data also present in abundance on social media. Through experiments we demonstrate the effectiveness of our method, achieving state-of-the-art performance in ADR mention extraction.

**Conclusion:**

In this study, we tackle the problem of labeled data scarcity for Adverse Drug Reaction mention extraction from social media and propose a novel semi-supervised learning based method which can leverage large unlabeled corpus available in abundance on the web. Through empirical study, we demonstrate that our proposed method outperforms fully supervised learning based baseline which relies on large manually annotated corpus for a good performance.

## Background

Social media is a useful platform to share health-related information due to its vast reach. This makes it a good candidate for public-health monitoring tasks, specifically for pharmacovigilance. We study the problem of extraction of Adverse-Drug-Reaction (ADR) mentions from social media, particularly from Twitter. Medical information extraction from social media is challenging, mainly due to short and highly informal nature of text, as compared to more technical and formal medical reports.

Current methods in ADR mention extraction rely on supervised learning methods, which suffer from labeled data scarcity problem. The state-of-the-art method uses deep neural networks, specifically a class of Recurrent Neural Network (RNN) which are Long-Short-Term-Memory networks (LSTMs) [1]. Deep neural networks, due to their large number of free parameters rely heavily on large annotated corpora for learning the end task. But in the real-world, it is hard to get large labeled data, mainly due to the heavy cost associated with the manual annotation. To this end, we propose a novel semi-supervised learning based RNN model, which can leverage unlabeled data also present in abundance on social media. Through experiments we demonstrate the effectiveness of our method, achieving state-of-the-art performance in ADR mention extraction.

Adverse-Drug-Reactions (ADRs) are a leading cause of mortality and morbidity in health care. In a study, it was observed that from a death count in the range of (44,000-98,000) due to medical errors, 7000 deaths occurred due to ADRs [2]. Postmarket drug surveillance is therefore required to identify such potential adverse reactions. The formal systems for postmarket surveillance can be slow and under-efficient. Studies show that 94% ADRs are under-reported [3].

Social media presents a useful platform to conduct such postmarket surveillance, given the large audience and vast reach of such platforms. Such platforms have been used for real-time information retrieval and trends tracking, including digital disease surveillance system [4]. A recent study shows that Twitter has 3 times more ADRs reported than were reported through FDA. Out of 61,000 tweets collected, 4400 had mention of ADRs as compared to 1400 ADRs reported through FDA during the same time-period [5]. This makes Twitter a great source for building a real-time post-marketing drug safety surveillance system. However, information extraction from social media comes with its own set of challenges. Some of them are: **1)** Short nature of the text (Twitter has a 142 character limit), making the language more ambiguous. **2)** Sparsity of drug-related tweets **3)** Highly colloquial language as compared to more technical and formal medical reports.

Consider for example the tweets, ‘*Cymbalta, you’re driving me insane’; ‘@ <USER> Ugh, sorry. This effexor is not making me feel so awesome*’. In the first tweet, ‘*driving me insane*’ and in the second one, ‘*not making me feel so awesome*’ are ADR mentions which indicate some level of discomfort in the user’s body. These tweets clearly demonstrates how information extraction from social media suffers from above-mentioned problems.

Recent work in deep learning has demonstrated its superiority over traditional hand-crafted feature based machine learning models [6, 7]. However, due to a large number of free parameters, deep learning models rely heavily on large annotated datasets. In the real-world, it is often the case that labeled data is sparse, making it challenging to train such models. Semi-supervised learning based methods provide a viable alternative solution to this. These methods rely on a small labeled data and a large unlabeled data for training.

In this work, we present a novel semi-supervised Recurrent Neural Network (RNN) [8] based method for ADR mention extraction, which leverages a relatively larger unlabeled dataset. We demonstrate the effectiveness of our method through experimentation on an ADR mention annotated tweet corpus [9]. Our method achieves superior results than the current state-of-the-art in ADR extraction from Twitter. In summary, our main contributions are:

- We propose a novel semi-supervised sequence labeling method based on Long-Short-Term-Memory (LSTM) network [1] which are known to capture long-term dependencies better than vanilla RNNs.
- For the unsupervised learning part, we explore a novel problem of drug name prediction given the drug’s context from tweets. The goal is to predict the drug name which is masked, given it’s context in the tweet.
- For supervised learning, we explore different word embedding initialization schemes and present results for the same.
- We demonstrate that by training a semi-supervised model, ADR extraction performance can be improved significantly as compared to current methods.
- On the Twitter dataset with ADR mentions annotated [9], our method achieves an F-score of 0.751 outperforming the current state-of-the-art method by ∼3%.

## Related work

The task of ADR mention extraction falls under the category of sequence labeling problems. The state-of-the-art method for solving sequence labeling problems is the Conditional Random Field (CRF) [10]. ADRMine [11], is a CRF-based model for ADR extraction task. It uses a variety of hand-crafted features, including word context, ADR lexicon, POS-tag and word embedding based features as input to CRF. The word embedding based features are trained on a large domain-specific tweet corpus. The problem with the above-mentioned approach is its dependency on hand-crafted features, which is time and effort consuming. A Long-Short-Term-Memory (LSTM) network based model is proposed [9] to get around this problem. Instead of using hand-designed features, word embedding based features are passed to a Bi-directional LSTM model which is trained to generate a sequence of labels, given the input word sequence. State-of-the-art results are achieved, surpassing CRF-based ADRMine results.

Some recent work also focuses on the problem of Adverse-Drug-Event (ADE) detection [12, 13]. Here the goal is to identify whether there is occurrence of an adverse-drug-reaction event or not. This is closely related to the problem addressed in the paper, with the difference being that instead of identifying where in the text adverse-drug-reactions are mentioned, we have to *identify* whether there is an occurrence or not.

## Method

### ADR-mention extraction using semi-supervised Bi-directional LSTM

In this section, we present our approach for ADR extraction. Our method is based on a semi-supervised learning method which operates in two phases: **1) Unsupervised learning:** In this phase, we train a Bidirectional-LSTM (bi-LSTM) [8] model to predict the drug name given its context in the tweet. As training data for this task, we select tweets with exactly one mention of any prescription drug. Since we already know the drug name beforehand, it doesn’t need any annotation effort. **2) Supervised learning:** In this phase, we use the trained bi-LSTM model from phase 1 and (re)train it to predict the sequence of labels, given the tweet text.

### Unsupervised learning

For this phase, we attempt a novel task of drug name prediction from its context in a tweet. This stage works as follows:

- Given a tweet, identify the drug name mentions using a curated drug name lexicon.
- Once drug names are identified in the tweet, replace all drug name mentions with a single dummy token (<*DRUG*>).
- In the spirit similar to the Continuous Bag of Words (CBOW) model of the well-studied word2vec model [14], we use the context of the masked drug name in the tweet as input to predict the actual drug name.

The intuition for the unsupervised stage is that the network will learn the context around the drug which will contains both positive and negative ADR mentions. Since the sequence classification models rely on context for classification, a rich knowledge of the context can serve as a good prior. Consider for example an ADR mention tweet, “*Since last week,lamotrigine causing steven johnson syndrome*”. As first step of the unsupervised stage, drug name mention “lamotrigine” is identified through a curated drug name lexicon. It is then masked with a dummy token. The transformed tweet is, *“Since last week, <DRUG> causing steven johnson syndrome”*. Now, for the drug name prediction task’s training, the input is presented to the network in the form: {**tweet text**, **target drug name**}, where tweet text is the drug name masked tweet and the target drug name is “lamotrigine” in this case. Due to the common misspelling errors on social media, people may refer to drug names differently and potentially with spelling errors. For such cases, we rely on drug name matching systems which can handle noisy drug mentions to identify drug names from novel tweets [15].

For creating training data, we use a large collection of tweets with exactly one mention of the drug name in them. Since we are predicting the drug name from a tweet which is already present in it, in order to avoid the network to learn a trivial function which maps drug name in input to drug name in output without considering the context in account, we mask the drug name in the tweet with a dummy token. This will force the network to look at the context as well. For feature-extraction, we use a bi-LSTM based model. The model takes as input, a sequence of continuous word vectors as input and predicts a corresponding sequence of word vectors as output. The equations governing the dynamics of LSTMs are defined as follows:

1 $$ \begin{aligned} \vec g^{u} & = \sigma\left(W^{u} * \vec h_{t-1} + I^{u} * \vec {\mathbf{x}}_{t}\right) \\ \vec g^{f} & = \sigma\left(W^{f} * \vec h_{t-1} + I^{f} * \vec {\mathbf{x}}_{t}\right) \\ \vec g^{c} & = \tanh\left(W^{c} * \vec h_{t-1} + I^{c} * \vec {\mathbf{x}}_{t}\right) \\ \vec m_{t} & = \vec g^{f} \odot + \vec g^{u} \odot \vec g^{c} \\ \vec g^{o} & = \sigma\left(W^{o} * \vec h_{t-1} + I^{o} * \vec {\mathbf{x}}_{t}\right) \\ \vec h_{t} & = \tanh\left(\vec g^{o} \odot \vec m_{t-1}\right) \end{aligned}  $$

here *σ* is the logistic sigmoid function, **W**^*u*^,**W**^*f*^,**W**^*o*^,**W**^*c*^ are recurrent weight matrices and **I**^*u*^,**I**^*f*^,**I**^*o*^,**I**^*c*^ are projection matrices. In a conventional LSTM, the sequence is read from left to right. In bi-LSTM, two sequence directions are considered, one from left to right and the other one opposite to it. The final hidden layer’s activation is the concatenation of vectors from both directions. Mathematically,

2 $$ \mathbf h_{t} = \left[\vec h_{t} ; \overleftarrow{h}_{t}\right]  $$

To generate the final representation of the tweet, average-pooling is applied over all hidden state vectors.

3 $$ \mathbf h = \sum_{t=1}^{T} \mathbf h_{t}  $$

where T is the maximum time-step. Finally a softmax transformation is applied to generate a probability distribution over all drug names followed by a categorical cross-entropy loss.

### Supervised sequence classification

For this phase, we take the bi-LSTM model trained from the previous phase and use it in a setup similar to state-of-the-art [9]. At each time-step of the sequence, a softmax layer is applied which gives a probability distribution over sequence labels. Formally,

4 $$ \mathbf y_{t} = softmax(\mathbf{W} \mathbf{h} + \mathbf{b}) \\  $$

here **W** and **b** are weight matrices for the softmax layer. The final loss for the sequence labeling is sum of categorical cross-entropy loss at each time-step defined as follows:

5 $$\begin{array}{@{}rcl@{}} L_{\text{ADR}} = - \sum_{t=1}^{n} \sum_{i=1}^{d_{l}} \hat{y_{t_{i}}} \log y_{t_{i}} \end{array} $$

where $\hat {y_{t}}$ is the one-hot representation of the actual label at time-step *t*, and $y_{t_{i}}$ is the ith component of the network prediction *y*_*t*_ as described above.

The hidden state **h** and the parameters **W**^*u*^, **W**^*f*^, **W**^*o*^, **W**^*c*^, **I**^*u*^, **I**^*f*^, **I**^*o*^, **I**^*c*^ are shared during training of both phases.

### Overall system pipeline

**Fig. 1:**
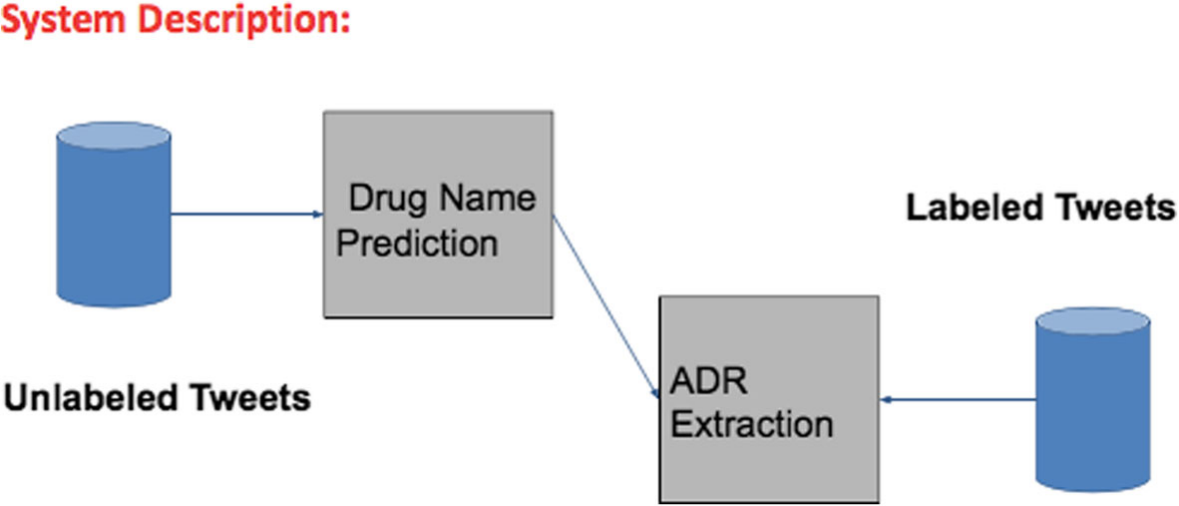
Overall System Diagram System diagram illustrating the connection between unsupervised learning and supervised learning phase

The overall system pipeline is described in Fig. 1. The first stage of the pipeline involves training the Bi-LSTM model on the unsupervised drug name prediction task. The weights updated during the training for the unsupervised stage are saved. For the second stage in the pipeline (supervised ADR extraction), a bi-LSTM model is initialized with the weights saved from the unsupervised stage and is trained for the supervised ADR extraction task. During prediction (testing) stage, the network weights obtained as result of training on both tasks are used.

## Results

### Dataset description

For the supervised-learning phase, we use the Twitter dataset annotated with ADR mention which were collected during the period of 2007-2010. Tweets were collected using 81 drug names as keyword search terms, which can be downloaded from [16]. In the original dataset, a total of 960 tweets are annotated with word-level ADR mentions. Twitter’s search API’s license prohibits the sharing of actual tweet content, so the released tweets dataset contains tweet ids along with the mention annotation. Out of the total of 960 tweets released as part of the original dataset, we were able to recover a total of 645 tweets text using Python library tweepy [17]. The rest of the tweets were not available, mostly due to deletion by users. According to the given train-test split in the baseline paper, 470 tweets are used for training and 170 tweets are used for testing.

For generating the unlabeled dataset (for the drug name prediction task), we used the Twitter’s search API [18] with the drug names used in the original study as keyword search terms [16].

The drug names used as keywords for searching related tweets are: **humira, dronedarone, lamictal, pradaxa, paxil, zoledronic acid, trazodone, enbrel, cymbalta, quetiapine**. We crawled the tweets over a period of two months. For simplicity, we removed the tweets with more than one drug mentions, resulting in a total of 0.1 Million tweets.

### Implementation details

We use Keras [19], a popular deep learning python library for implementation. For text pre-processing on both supervised and unsupervised corpus, we applied the following steps:

- Normalizing HTML links and user-mentions:We replaced all HTML link mentions with the token “ <*LINK*>”. Similarly, we replaced all user handle mentions (for ex. @JonDoe) with the token “ <*USER*>”.
- Special Character Removal: We removed all punctuations and special symbols like ‘#’ from tweets.
- Emoticons Removal: We removed all emoticons, in general all non-ascii characters which are special types of emoticons.
- Stop-word and rare words removal: We removed all stop-words and set the vocabulary size to top-15000 most frequent words in the corpus.

We used the word2vec [14] embeddings trained on a large generic Twitter corpus [20] as input to the model. Word vector dimension is set to 400. Bi-LSTM parameters are set to the best reported setting from [9], with hidden unit’s dimension equal to 500. For training the supervised model, we use the adam optimizer [21] with batch-size equal to 1 and for training the unsupervised model, we used the batch adam optimizer [21] with batch-size set to 128 empirically. The supervised model was trained for a total of 5 epochs, and the unsupervised model was trained for 30 epochs.

To convert the ADR extraction problem into a sequence labeling problem, we need to assign the annotated entities with appropriate tag representations. We follow the IO encoding scheme, where each word belongs to either of the following categories: **(1)** I-ADR (inside ADR) **(2)** I-Indication (inside Indication), where indication is the symptom indicating presence of some disease caused by the drug **(3)** O (Outside any mention) **(4)** <*PAD*> (if the word is padding token). An example tweet annotated with IO-encoding: *@BLENDOS*_*O*_*Lamictal*_*O*_*and*_*O*_*trileptal*_*O*_*and*_*O*_*seroquel*_*O*_*of*_*O*_*course*_*O*_*the*_*O*_*seroquel*_*O*_*I*_*O*_*take*_*O*_*in*_*O*_*severe*_*O*_*situations*_*O*_*because*_*O*_*weight*_*I*−*ADR*_*gain*_*I*−*ADR*_*is*_*O*_*not*_*O*_*cool*_*O*_. It should be noted that similar to the baseline [9], we report the performance on the ADR label only. This is because the number of Indication annotations are very less in number:**45 in training, 16 in testing**.

### Evaluation

For performance evaluation, we use approximate-matching [22], which is used popularly in biomedical entity extraction tasks [9, 11]. Approximate matching considers a predicted ADR span correct if it overlaps with one or more actual ADR spans. For instance, given the tweet *“The Seroquel gave me lasting sleep paralysis”* with the true ADR span *“sleep paralysis”*, predicted spans of *“lasting sleep paralysis”* or simply *“paralysis”* are counted as correct.

We report the F1-score, Precision and Recall computed using approximate matching as follows:

6 $$ \text{Precision (P)} = \frac{\text{\#ADR approximately matched}}{\text{\#ADR spans predicted}}  $$

7 $$ \text{Recall (R)}= \frac{\text{\#ADR approximately matched}}{\text{\#ADR spans in total}}  $$

8 $$ \text{F1-score}= \frac{2PR}{P+R}  $$

Table 1 presents the results of our approach along with comparisons. Since the number of tweets used for training and testing differs from the one used in baseline [9], we re-ran their model using the source-code released by them [23]. It should be noted that the original model used RMSProp [24] as an optimizer, so for a fair comparison with our method, we also report the baseline results with optimizer as adam instead of RMSProp. Replacing RMSProp with adam, although gives an improvement over the original baseline, it still underperforms our method. Our approach gives state-of-the-art results, with an improvement of ∼3% F1 over the original baseline and an improvement of 1.88% F1 over the re-implemented baseline.

**Table 1:** Performance of various deep neural network methods on ADR extraction task

Method	F1-Score	Precision	Recall
**Baseline** [9]	0.729 ±0.027	0.695 ±0.109	**0.776 ±0.121**
Baseline (with adam optimizer)	0.737 ±0.308	0.707 ±0.096	0.774 ±0.08
Semi-Supervised ADR extraction	0.751 ±0.036*	0.731 ±0.035*	0.774 ±0.073

^⋆^Indicate statistical significant (*p*≤0.05) using paired t-tests compared to the baseline

Highlighted portions reflect the best results across the respective column

### Discussion

#### Effect of drug-mask

For the unsupervised learning phase, we select the task of drug name prediction given its context. In order to avoid the network learning a degenerate function which maps input drug name to output drug name, we mask all drug names in input with a single token. In order to verify this, we report the accuracy results without the drug-mask, i.e. with drug name included in the input. The result is presented in Table 2. It is clear that removing the drug mask from input degrades the end-performance by 0.535% in F-score. This further validates our claim that masking the drug names is effective.

**Table 2:** Performance comparison of Semi-Supervised bi-LSTM (SS-BLSTM) under different word embedding initialization settings and different unlabeled data settings. Results are reported averaged over 30 trials along with the std. deviation

Method	F1-Score	Precision	Recall
SS-BLSTM (with drug mask removed)	**0.747 ±0.037**	0.723 ±0.106	**0.780 ±0.108**
SS-BLSTM (with labeled tweets dictionary only)	0.745 ±0.039	**0.727 ±0.072**	0.769 ±0.097
SS-BLSTM (with GoogleNews [25] vectors)	0.736 ±0.031	0.708 ±0.095	0.774 ±0.118
SS-BLSTM (with medical embeddings)	0.673 ±0.021	0.642 ±0.089	0.716 ±0.118

Highlighted portions reflect the best results across the respective columns

#### Effect of embeddings and dictionary

We experiment with word embeddings trained on different corpora to observe its effect on performance. We experiment with embeddings trained on a part of Google News dataset, which consists of around 100 billion words [25]. It can be observed that using Google News corpus trained embeddings degrades the performance by 2.04% in F-score. This is due to the fact that these embeddings are trained on a large news corpus, which is grammatically more sound and formal than the colloquial social media language. Conceptually, the shift in the lexical data distribution of the news corpus as compared to tweets containing ADR causes the degradation in performance. We also experiment with word embeddings trained on a large medical-concept terms related tweet corpus [26, 27]. Intuitively, embeddings trained on similar domain (medical in this case) should perform better, but surprisingly it performs worst amongst all methods. The generic embeddings trained on large tweet corpus captures potentially large variation of semantics and linguistic properties of text and due to the free-style nature of writing on social media, this helps more than domain-knowledge, as captured by medical-domain trained embeddings.

We also experimented with a different vocabulary initialization. In our proposed formulation, we construct vocabulary from both unlabeled and labeled corpus, resulting in a larger vocabulary size. When experimented with a restricted vocabulary (only from labeled training data), we observe that the F1-score drops by 0.8%. This suggests the use of a larger vocabulary with more coverage in similar settings.

## Conclusions

We present a novel semi-supervised Bi-directional LSTM based model for ADR mention extraction. We evaluate our method on an annotated Twitter corpus. By leveraging a potentially large unlabeled corpus, our method outperforms the state-of-the-art method by ∼3% in F1-score.

We also demonstrate that word embeddings trained on a large domain-agnostic Twitter corpus performs better than more popular Google News Corpus trained word-embeddings and surprisingly even better than medical domain-specific word embeddings trained on tweets, which suggests that language structure and semantics is more important in downstream information extraction tasks, compared to domain knowledge.

In future, we plan to explore drug and side-effect (adverse-effect) mention relation extraction along with ADR extraction and seek to validate if both can be formulated in a multi-task learncing setup.
